# The importance of vector control for the control and elimination of vector-borne diseases

**DOI:** 10.1371/journal.pntd.0007831

**Published:** 2020-01-16

**Authors:** Anne L. Wilson, Orin Courtenay, Louise A. Kelly-Hope, Thomas W. Scott, Willem Takken, Steve J. Torr, Steve W. Lindsay

**Affiliations:** 1 Department of Vector Biology, Liverpool School of Tropical Medicine, Liverpool, United Kingdom; 2 Zeeman Institute and School of Life Sciences, University of Warwick, Coventry, United Kingdom; 3 Department of Tropical Disease Biology, Liverpool School of Tropical Medicine, Liverpool, United Kingdom; 4 Department of Entomology and Nematology, University of California Davis, Davis, California, United States of America; 5 Department of Plant Sciences, Wageningen University and Research, Wageningen, the Netherlands; 6 Department of Biosciences, Durham University, Durham, United Kingdom; Centers for Disease Control and Prevention, Puerto Rico, UNITED STATES

## Abstract

Vector-borne diseases (VBDs) such as malaria, dengue, and leishmaniasis exert a huge burden of morbidity and mortality worldwide, particularly affecting the poorest of the poor. The principal method by which these diseases are controlled is through vector control, which has a long and distinguished history. Vector control, to a greater extent than drugs or vaccines, has been responsible for shrinking the map of many VBDs. Here, we describe the history of vector control programmes worldwide from the late 1800s to date. Pre 1940, vector control relied on a thorough understanding of vector ecology and epidemiology, and implementation of environmental management tailored to the ecology and behaviour of local vector species. This complex understanding was replaced by a simplified dependency on a handful of insecticide-based tools, particularly for malaria control, without an adequate understanding of entomology and epidemiology and without proper monitoring and evaluation. With the rising threat from insecticide-resistant vectors, global environmental change, and the need to incorporate more vector control interventions to eliminate these diseases, we advocate for continued investment in evidence-based vector control. There is a need to return to vector control approaches based on a thorough knowledge of the determinants of pathogen transmission, which utilise a range of insecticide and non–insecticide-based approaches in a locally tailored manner for more effective and sustainable vector control.

## Author summary

Vector-borne diseases (VBDs) such as dengue, Chagas disease, human African trypanosomiasis (HAT), leishmaniasis, and malaria exert a huge burden of morbidity and mortality worldwide. The principal method by which these diseases are controlled is through vector control. The authors chart the history of vector control through time from elucidation of the transmission route of VBDs to the present day. Pre-1940 vector control relied heavily on environmental management and larval control based on a thorough understanding of pathogen transmission but was replaced by insecticide-based vector control, often deployed as a monotherapy. The authors call for increased political will and investment in vector control and a return to locally tailored vector control that draws on the entire toolbox of interventions available.

## Introduction

Vector-borne diseases (VBDs) are infections caused by pathogens that are transmitted by arthropods such as mosquitoes, triatomine bugs, blackflies, tsetse flies, sand flies, lice, and ticks. Dengue, Chagas disease, Japanese encephalitis, leishmaniasis, lymphatic filariasis (LF), malaria, and yellow fever threaten over 80% of the world’s population and disproportionately affect the poorest populations living in the tropics and subtropics [1]. Many of these VBDs are co-endemic, and it is estimated that more than half the world’s population live in areas where 2 or more VBDs are present [1]. VBDs contribute significantly to the global burden of disease, accounting for 17% of the global estimated burden of all infectious diseases [2]. Perhaps the best known VBD, malaria, is a major cause of morbidity and mortality, particularly in sub-Saharan Africa (SSA), with approximately half the world’s population predicted to be at risk of malaria (Table 1) [3]. Many VBDs are classified as neglected tropical diseases (NTDs), e.g., arboviral diseases like dengue and chikungunya, Chagas disease, human African trypanosomiasis (HAT), leishmaniasis, LF, and onchocerciasis [4]. The burden of NTDs is poorly understood, and until the last 5 to 10 years, these diseases have suffered from a lack of prioritisation and investment. Important aspects of NTD biology, epidemiology, and prevention remain inadequately understood. While the global numbers of deaths from vector-borne NTDs is lower than for malaria, vector-borne NTDs continue to cause high levels of morbidity and represent a significant public health burden; e.g., from 1990 to 2013, dengue cases increased nearly 450% globally [5]. Some zoonotic NTDs have an additional veterinary health burden [6, 7].

**Table 1:** Global burden of VBDs.

	Data source	Estimated cases worldwide in 2017 (thousands [95% CI])	Estimated global all-age DALYs in 2017 (thousands [95% CI])	Estimated all-age deaths worldwide in 2017 (thousands [95% CI])
**Malaria**	World Malaria Report 2018 [8]	219,000 (203,000–262,000)	Not stated	435
	Global Burden of Disease 2017 [6, 7, 9]	208,768 (170,214–257,506)	45,000 (31,700–61,000)	619.8 (440.1–839.5)
**Dengue**	Global Burden of Disease 2017 [6, 7, 9]	104,771 (63 759–158,870)	2,920 (1,630–3,970)	40.5 (17.6–49.8)
**CL and mucocutaneous leishmaniasis**		4,166.6 (3,560.7–4,992.8)*	264 (172–389)	-
**VL**		10.6 (8.2–16.5)*	511 (1.02–2,440)	7.5 (0.0–34.5)
**Yellow fever**		97.4 (28.0–251.7)	314 (67.2–900)	4.8 (1.0–13.8)
**Chagas disease**		6,197.0 (5,248.5–7,243.9)*	232 (210–261)	7.9 (7.5–8.6)
**HAT**		4.9 (1.3–19.8)*	79.0 (15.4–287)	1.4 (0.3–4.9)
**LF**		64,623.4 (59,178.2–70,866.1)*	1,360 (752–2,160)	-
**Onchocerciasis**		20,938.1 (12,882.3–37,227.7)*	1,340 (639–2,370)	-
**Trachoma**		3,818.9 (2,842.6–5,135.2)*	303 (202–425)	-
**Zika virus disease**		2,232.2 (1,659.6–3,097.6)	2.24 (1.27–4.66)	0.0 (0.0–0.1)

*Prevalence.

**Abbreviations:** CL, cutaneous leishmaniasis; DALY, disability-adjusted life year; HAT, human African trypanosomiasis; LF, lymphatic filariasis; VBD, vector-borne disease; VL, visceral leishmaniasis

Vector control is the principal method available for controlling many VBDs—both historically and today. Moreover, for some diseases, such as dengue (a vaccine is licensed but is not widely used due to safety concerns [10]), chikungunya, Zika, and West Nile disease, vector control is currently the only method available to protect populations. Vector control aims to limit the transmission of pathogens by reducing or eliminating human contact with the vector. A wide range of vector control tools exist, which can be broadly classified into chemical- and non–chemical-based tools (Table 2). Tools targeting immature vectors can act by killing the immature stages (e.g., chemical or biological larvicides and predator species) or by removing suitable aquatic habitats (e.g., habitat modification or manipulation). Tools targeting the adult vectors function by killing the vector (e.g., indoor residual spraying [IRS], space spraying) and/or reducing vector contact (blood-feeding success) with human and/or animal reservoir hosts (e.g., topical repellents, house screening, insecticide-treated bed nets [ITNs], insecticide-treated dog collars). There are also several novel vector control tools under development, e.g., genetic manipulation of mosquitoes, bacterial infection of vectors (e.g., *Wolbachia*), and insecticide-treated eave tubes (Box 1).

**Box 1. Examples of novel vector control interventions being developed**

**Gene drive**

Gene drive is a method of genetic modification that can be used to spread favourable traits through interbreeding populations of malaria mosquitoes [12]. The gene drive allows genes to spread through populations in a self-sustaining manner, even if they confer a fitness cost. The technique can be used for population replacement (reducing the ability of mosquitoes to transmit a pathogen) or population suppression (reducing the size of the vector population by, e.g., reducing fertility of females or biasing the sex ratio towards males). The ‘Target Malaria’ group started initial work in Burkina Faso, in preparation for releases of gene drive mosquitoes.

**Wolbachia**

*Wolbachia* is a genus of bacteria that naturally infect some insect species but are not normally found in *Aedes* mosquitoes. Introduction of *Wolbachia* into *Aedes* mosquitoes reduces transmission of dengue and other arboviruses to people [13]. Mosquitoes carrying *Wolbachia* are released by field teams and mate with the wild mosquito population, and over time the percentage of mosquitoes carrying *Wolbachia* increases. The ‘World Mosquito Program’ is conducting a randomised controlled trial and programmatic evaluations in several countries worldwide (e.g., [14, 15]).

**Spatial repellents**

Spatial repellents are chemicals that prevent a vector from entering a space occupied by a potential human host to reduce encounters between the vector and the host [16]. They are typically used against malaria or *Aedes* mosquitoes and can be used indoors or outdoors, where they may serve as a useful tool in combating outdoor transmission of malaria. Spatial repellents exist in a number of formats, including coils, passive emanators, and impregnated fabric. Although several small trials of spatial repellent coils have been performed [17, 18], spatial repellents are not yet recommended for disease prevention. Results from randomised controlled trials for malaria and dengue are currently being analysed. A research programme was recently funded by UNITAID to carry out a second efficacy evaluation of spatial repellent passive emanators against malaria, and a second trial is being considered for dengue.

**Eave tubes**

Eave tubes are small plastic tubes with insecticide-laden electrostatic netting that are inserted into the house wall, below the roof [19]. Mosquitoes are lured to the house by host odours emanating through the eave tubes and are killed after contacting the insecticide-treated netting. Eave tubes are being tested in a large randomised-controlled trial in Côte d’Ivoire, which includes other house improvements, including eave closure and window screening [20].

**Table 2:** Categories and examples of vector control methods [11].

**Chemical**	Immature	Chemical larvicides	Contact pesticides affecting insect nervous system (e.g., temephos) or endocrine system (insect growth regulators, e.g., pyriproxyfen)
	Adult	ITNs	Pyrethroid-treated ITNs or combination ITNs (e.g., pyrethroid plus synergist piperonyl butoxide) for malaria, LF, and leishmaniasis control
		Insecticide-treated materials for personal protection	Insecticide-treated clothing for workers and mobile populations
		IRS	Spraying of residual insecticides (typically either pyrethroids, carbamates, or organophosphates) indoors for malaria and *Aedes*-borne disease control
		Space spraying	Aircraft, vehicle or hand-held space spraying for dengue epidemic and other *Aedes*-borne disease control
		Insecticidal treatment of habitat	Focal, perifocal, ground, or aerial insecticide spraying
		Insecticide-treated cattle	Pour-on or spot-on pyrethroids for control of tsetse
		Insecticide-treated traps and targets	Targets for control of HAT and insecticide-treated adulticidal oviposition traps for *Aedes*-borne diseases
		Topical repellent	Chemicals (e.g., N,N-diethyl-meta-toluamide [DEET], picaridin) applied to the skin to reduce vector biting
		Spatial repellent	Transfluthrin/metafluthrin passive emanators or coils
**Nonchemical**	Immature	Microbial larvicides	*Bacillus thuringiensis* var. *israelensis*, *B*. *sphaericus*
		Predator species	Predatory fish or invertebrates
		Habitat modification, i.e., a permanent change of land and/or water	Drainage of surface water, land reclamation and filling, and coverage of large water storage containers (or complete coverage of water surfaces) with a material that is impenetrable to mosquitoes, such as expanded polystyrene beads
		Habitat manipulation, i.e., a recurrent activity	Water-level manipulation, exposing habitats to the sun (depending on the ecology of the vector), flushing of streams, drain clearance, and source reduction, including rubbish disposal and regular emptying and cleaning of domestic containers (e.g., flowerpots, animal drinking water troughs)
		Regulatory measures	Removal of man-made aquatic habitats and appropriate waste disposal
	Adult	House improvement and screening	Closing eaves, door and window screening
		Removal trapping	Solar-powered mosquito trapping system for malaria control and sticky adulticidal oviposition traps for *Aedes*-borne diseases

**Abbreviations:** HAT, human African trypanosomiasis; IRS, indoor residual spraying; ITN, insecticide-treated bed net; LF, lymphatic filariasis

**Fig 1:**
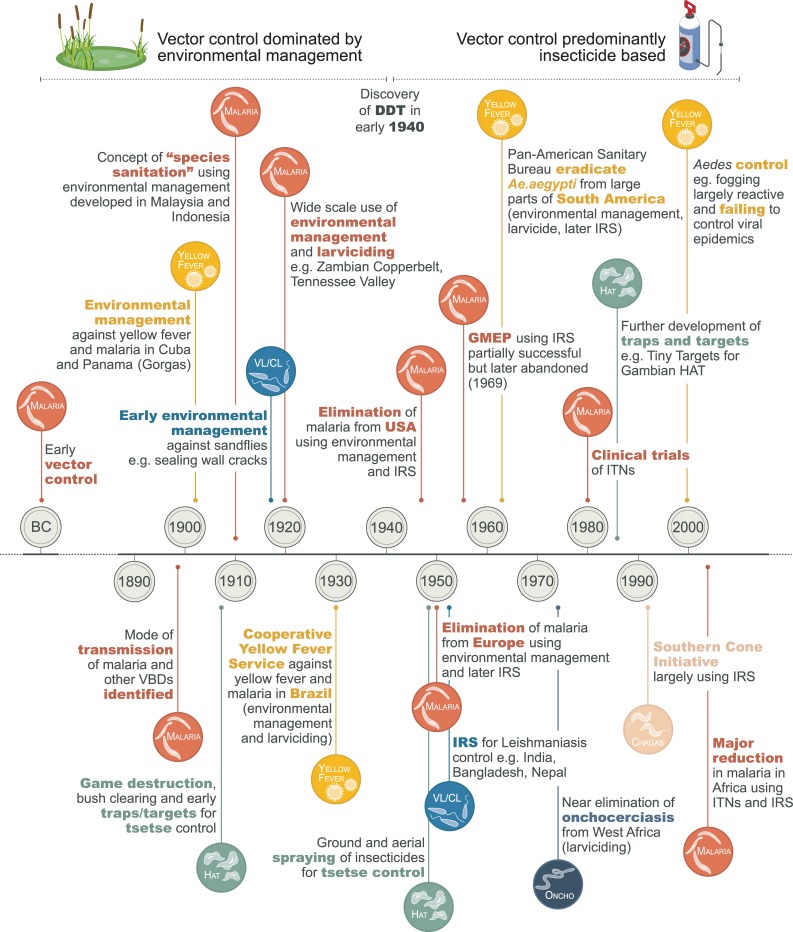
A short history of vector control. CL, cutaneous leishmaniasis; DDT, dichlorodiphenyltrichloroethane; GMEP, Global Malaria Eradication Programme; HAT, human African trypanosomiasis; IRS, indoor residual spraying; ITN, insecticide-treated bed net; VBD, vector-borne disease; VL, visceral leishmaniasis.

Our goal was to review the history of vector control from the 1800s to the present day, highlighting what tools and approaches were adopted and the impacts they had on vectors and infection and disease. This Review focuses on the main VBDs: *Aedes*-borne viruses, Chagas disease, HAT, leishmaniasis, LF, malaria, and onchocerciasis. Malaria is the dominant focus of the Review given the high burden of morbidity and mortality and well-documented history of thinking on malaria vector control [21, 22]. The manuscript uses some terminology that is often misused in the public health literature and may require definition: ‘control’ refers to deliberate efforts to reduce disease incidence, prevalence, morbidity, or mortality to a locally acceptable level; ‘elimination’ refers to interruption of local transmission requiring continued efforts to prevent reestablishment of transmission; and ‘eradication’ refers to permanent reduction to zero of the worldwide incidence of infection as a result of deliberate efforts, so that intervention measures are no longer needed [23]. A summary of the programmes and campaigns discussed is given in Fig 1 and Table 3. The World Health Organization (WHO) calls for effective, locally adapted, and sustainable vector control in its recent Global Vector Control Response (GVCR) 2017–2030 strategy [2]. Increasing our understanding of the different types of vector control tools that were used in the past, what settings they were used in, and their impacts on vectors and disease can hopefully help us to select and promote new and existing vector control methods to sustainably reduce the burden and threat of VBDs. We conclude by drawing out key lessons from the history of vector control and identifying how these can be applied now and in the future.

**Table 3:** Historical overview of notable vector control programmes and their effects.

Date	Location	Programme	Disease	Vector species targeted	Vector control methods implemented	Effects observed	Reference
Late 1800s	East Africa	Efforts led by colonial powers	HAT	*Glossina*	Bush clearance, game destruction, trapping of tsetse	Decline in tsetse populations	[24–27]
1901–1920	Malaya (now Malaysia)	Efforts led by Sir Malcolm Watson	Malaria	*Anopheles umbrosus* *An*. *maculatus*	Draining marshes, subsoil drainage, filling water bodies, tree clearing, relocating housing	Reduction in malaria	[28]
1901–1912	Cuba	Efforts led by Gorgas and Le Prince, taken over by local authorities after 1904	Yellow fever and malaria	*Aedes aegypti* *An*. *albimanus*	Yellow fever: house inspection and destruction/oiling of containers, fines if cisterns not covered, and isolation of patients with screening and netting, and fumigation of their premises Malaria: drainage, filling or oiling of standing water, ditching, cutting vegetation at edges of ponds/streams, larvivorous fish and oiling of wells, restricting animal grazing during wet season, intermittent short-duration flooding of watercress beds (versus constant flooding)	Reduction in yellow fever and malaria	[29]
1904–1913	Panama Canal	Efforts led by Gorgas and Le Prince	Yellow fever and malaria	*An*. *albimanus* *An*. *tarsimaculata* *Ae*. *aegypti*	Yellow fever: house screening, premise and container inspections, destruction or treatment of containers with oil/larvicide Malaria: house screening, clearing water bodies, draining or filling standing water, installing drains, removing jungle, larviciding using oil or Paris Green	Reduction in malaria and yellow fever	[29] [30]
1920–1935	Indonesia	‘Species sanitation’ led by N. H. Swellengrebel	Malaria	*An*. *ludlowi* (now *An*. *sundaicus*) *An*. *aconitus* *An*. *maculatus*	Environmental management, e.g., filling and draining of ponds, maintaining and flushing drains, planting trees	Reduction in malaria	[31, 32]
1930–1962	Italy	Italian antimalarial campaign ‘bonfica integrale’ under Mussolini	Malaria	*An*. *labranchiae*	Draining of Pontine marshes, house screening, community education and mobilisation, larviciding using Paris Green, clearing canals and ditches, DDT aerial spraying (after 1946)	Malaria eradication	[33–35]
1930–1942	Brazil	Cooperative Yellow Fever Service directed by Fred Soper	Yellow fever and malaria	*Ae*. *aegypti* *An*. *gambiae*	Yellow fever: container inspections, oiling/larviciding of aquatic habitats, sanitary legislation enforced by monetary fines Malaria: larviciding with Paris Green, house spraying with short-acting pyrethroids	Elimination of *Ae*. *aegypti* from many areas, reduction in yellow fever; elimination of *An*. *gambiae* from northeast Brazil	[36]
1929–1950	Copperbelt, Zambia	Roan Antelope Copper Mine and others, including Nchanga Consolidated Copper Mines, Rhokana Corporation, and Mufulira Cooper Mines	Malaria	*An*. *gambiae* *An*. *funestus*	Vegetation clearance along river and tributaries, modification of river boundaries and removal of man-made obstructions, draining flooded areas and swamps, oiling of larval habitats, and house screening	At Roan Antelope Mine, reduced malaria-related mortality and morbidity by 70%–95% within 3–5 years	[37] [38]
1933–1950	Tennessee Valley, US	TVA	Malaria	*An*. *quadrimaculatus*	Regulation of water levels in the lakes, shoreline improvements such as deepening or diking and draining, larviciding, and later (to a limited extent) house improvement, DDT aerial spraying, and IRS using DDT	Virtual malaria elimination	[39, 40]
1942–1943 (World War II)	Guadalcanal, Solomon Islands	US Navy, Malaria Control Unit ‘Cactus’	Malaria	*An*. *farauti*	Oiling of swamps, fumigation of planes and huts, relocation of plantation workers, ITNs, topical repellents, atabrine prophylaxis	Reduction in malaria cases	[41, 42]
1942–1945	Upper Egypt	Species (*An*. *gambiae*) eradication	Malaria	*An*. *gambiae*	Larviciding with Paris Green (later Malariol due to supply issues); pyrethrum house spraying; residual spraying of boats, planes, trains, automobiles with pyrethrum (later DDT)	Massive reductions in malaria cases (10,193 cases in 1942, to 59 in 1946); eradication of *An*. *gambiae*	[43]
1947–1951	Southeast USA	US National Malaria Elimination Programme	Malaria	*An*. *quadrimaculatus* *An*. *freeborni*	IRS with DDT, larviciding using Paris Green, deepening or diking and draining of water bodies, lining canals with concrete	Free of malaria as a significant public health problem in 1949	[44]
1955–1969	Worldwide	Global Malaria Elimination Programme	Malaria	Varied depending on location	IRS with DDT and other residual insecticides	Elimination from some regions, but unsuccessful elsewhere	[45, 46]
1947–1962	South and Central America and Caribbean	Pan-American Sanitary Bureau	Yellow fever	*Ae*. *aegypti*	Container inspections, oiling of aquatic habitats, later perifocal spraying of DDT in water containers and nearby walls	Vector eradicated from large parts of South America	[47, 48]
1951–1980	China	National visceral leishmaniasis control programme	VL	*Phlebotomus chinensis* *P*. *longiductus* *P*. *wui* *P*. *alexandri*	IRS of houses and animal shelters using DDT, and elimination or topical deltamethrin treatment of dogs	Massive reduction in case incidence from 94/100,000 in 1950 to approximately 0.03/100,000 by 1980	[49]
1950s–1970s	Peruvian Andes	Gains as a result of Global Malaria Elimination Programme	CL	*Lutzomyia peruensis* *Lu*. *verrucarum* *Lu*. *ayacuchensis*	IRS with DDT	Decrease in cases but resurgence once IRS was stopped	[50]
1973–1991	Botswana	Tsetse control programme	HAT	*G*. *morsitans centralis*	Aerial spraying of insecticides; deltamethrin-treated targets to stop reinvasion of tsetse	Eliminated tsetse and HAT	[25, 51]
1970s–present	Solomon Islands and Papua New Guinea	Malaria Elimination Programme Pacific Programme for the Elimination of Lymphatic Filariasis (PacELF) post 1999	LF	*An*. *farauti* *An*. *koliensis* *An*. *punctulatus*	Solomon Islands: IRS using DDT Papua New Guinea: IRS with DDT, later mosquito nets (untreated), and since 2005 long-lasting ITNs	Elimination from Solomon Islands by late 1970s, and near elimination from PNG (ongoing)	[52–54]
1974–2002	West Africa	Onchocerciasis Control Programme (OCP) and to a lesser extent African Programme for Onchocerciasis Control (APOC)	Onchocerciasis	*Simulium* spp.	Larviciding	Near elimination of river blindness from West Africa	[55, 56]
1991–present	Argentina, Bolivia, Brazil, Chile, Paraguay, and Uruguay	Southern Cone Initiative (SCI)	Chagas disease	*Triatoma infestans* and other species	IRS, house improvements, and community education	Decline in indoor infestation and disease incidence	[57–59]
1953–present	India, Nepal, Bangladesh	Visceral Leishmaniasis Elimination Programme–Memorandum of Understanding between countries signed 2005 (previous gains as a result of Malaria Eradication Programme)	VL	*P*. *argentipes*	IRS using DDT in homes and animal shelters	Decline in cases in 3 countries from 77,000 in 1992 to 6,000 in 2016	[60]
1915 (California)–present	US	Mosquito abatement districts	*Aedes*-borne diseases	*Aedes* and nuisance mosquitoes	Predominantly larval control	Prevention of local *Aedes*-borne virus transmission, e.g., no locally transmitted Zika in US states	[61]
2000–present	SSA	Campaign to eliminate HAT (numerous donors, research institutions, and implementing partners)	HAT	*Glossina* spp.	Screening and treatment, traps and targets (‘Tiny Targets’), insecticide-treated cattle	25,841 cases of *Trypanosoma brucei gambiense* HAT in 2000 to 2,110 in 2016 and from 709 *Trypanosoma brucei rhodesiense* HAT cases in 2000 to 54 in 2016	[62]
2000–present	SSA	Scale-up of ITNs and IRS	Malaria	*An*. *gambiae* and other species	ITNs and IRS	ITNs responsible for 68% of 663 million clinical cases averted from 2000 to 2015	[63]

**Abbreviations:** CL, cutaneous leishmaniasis; DDT, dichlorodiphenyltrichloroethane; HAT, human African trypanosomiasis; IRS, indoor residual spraying; ITN, insecticide-treated bed net; LF, lymphatic filariasis; SSA, sub-Saharan Africa; TVA, Tennessee Valley Authority; VL, visceral leishmaniasis

## A brief history of vector control

### Elucidation of the transmission route of VBDs and early vector control

In the 1890s and early 1900s, the transmission route of Chagas disease, HAT, LF, malaria, and yellow fever were elucidated, primarily in response to the germ theory of Pasteur and Koch and advancements in microscopy [64–69]. The first VBD of humans to be identified was malaria when in 1897, ground-breaking work by Sir Ronald Ross showed that *Anopheles* mosquitoes transmitted malaria parasites [66]. However, vector control had been taking place prior to this, mainly due to an awareness of the connection between fevers and proximity of swamps and marshes. For example, historical reports from Greek (circa 550 B.C.) and Roman times describe large drainage schemes and reductions in ‘plague’ and fever [70, 71]. There are reports of the use of mechanical vector control methods, such as sleeping in high buildings, where mosquitoes were unable to fly due to wind; the use of bed nets in Egypt (as noted by Herodotus 484–425 B.C.) and by the Romans; and the use of bed curtains, as noted by Marco Polo during his travels to India in the 13th century [70]. Sanitary measures helped to control yellow fever in the US in the late 1700s, including cleaning sewers and pumping bilge water out of ships, which was practiced in Philadelphia during a large outbreak in 1793 when refugee ships from Saint Dominigue (now Haiti) inadvertently brought in the *Ae*. *aegypti* vector [72, 73].

### Environmental management as the primary tool for control of VBDs

Prior to the use of insecticides, VBD control generally relied on understanding local vector behavioural ecology and tailored environmental control, although these were often labour intensive. Following Ross’s discovery and pioneering work on quantitative epidemiology of VBDs, the focus of malaria control was on elimination of anopheline vectors, primarily by changing the aquatic habitats where the vectors developed. There was also a focus on housing improvements such as screening of doors and windows. The first trial of a malaria intervention was carried out by Angelo Celli among railway workers in Italy in 1899–1900 [74–76]. The combined intervention of housing screening, whitewashing internal walls, burning special powders (probably pyrethrum), and protective clothing was highly successful. Celli found that 92% of the families left unprotected contracted malaria compared to only 4% in the intervention group.

Environmental management flourished in both Malaya (now Malaysia) and Netherlands East Indies (now Indonesia) in the early 1900s. British doctor Sir Malcolm Watson joined the Malayan Medical Service in 1900, where he led vector control efforts against malaria based on an understanding of the ecology of local vectors [28]. Drainage of aquatic habitats controlled malaria in 2 coastal towns in the state of Selangor and allowed resumption of port development. Later, Watson’s work expanded to lowland areas—where he controlled *An*. *umbrosus* by clearing the forest within 0.8 km of plantation labourer houses so that water bodies were exposed to the sun—and in 1909 to hilly regions, where subsoil drainage was successful against *An*. *maculatus*. Watson also oversaw successful drainage schemes in Singapore in 1911, where local malaria transmission was practically eliminated [28]. The Dutch zoologist Nicolaas Swellengrebel was inspired by Watson and set out to replicate the work throughout the Indonesian archipelago between 1920 and 1935, terming his methods ‘species sanitation’ [31]. Swellengrebel aimed to control malaria primarily through environmental management, such as filling in or draining ponds and lagoons, maintaining and flushing drains, or planting shade trees depending on the vector species present and its ecology.

The Pontine marshes, near Rome, Italy, suffered from high malaria transmission for over 1,000 years. Large-scale drainage from 1930 onwards was highly successful in malaria control against the Italian malaria vector *An*. *labranchiae* [34]. This was done as part of the three-pronged ‘bonifica integrale’ campaign, or ‘bonification’, instigated by Mussolini. The campaign included agricultural improvements—e.g., draining swamps to increase agricultural land; hygienic measures, such as building sturdy, well-screened brick housing; and quinine distribution.

Another excellent example of environmental management for malaria control was in the Zambian Copperbelt during the 1920s and 1930s [37, 38, 77]. Control measures launched at the Roan Antelope Copper Mine in 1929—including vegetation clearance, modification of river boundaries, draining swamps, oil application to open water bodies, and house screening—were highly effective in targeting *An*. *gambiae* and *An*. *funestus* [37]. In 3 to 5 years, malaria-related mortality and morbidity declined by 70%–95%, and over a 20-year period, the programme averted an estimated 4,173 deaths and 161,205 malaria attacks.

Environmental management was also being implemented on a large scale in the southern US. The Tennessee Valley Authority (TVA) was set up in 1933 to exploit the Tennessee River's potential for hydroelectric power and improve the land and waterways for agricultural development of the region [39, 40]. At the time, the region was highly endemic for malaria, and the creation of artificial lakes would have exacerbated the problem. Vector control tools implemented included regulation of water levels in lakes, shoreline improvements such as deepening or diking and draining, larviciding, house screening, and later dichlorodiphenyltrichloroethane (DDT) spraying. Massive reductions in malaria were seen, and the disease was essentially eliminated from the area by the late 1940s.

Control of yellow fever in the Americas at the start of the 20th century was heavily reliant on environmental management. At this time, the US had taken control of Cuba following the end of the Spanish American war, but outbreaks of yellow fever and malaria were taking the lives of many US soldiers [78]. Following confirmation that *Ae*. *aegypti* was the yellow fever virus vector, in 1901 Major William Gorgas, Chief Sanitary Officer, was asked to initiate a programme for the elimination of *Ae*. *aegypti*, work he carried out with Joseph Le Prince [29]. The programme was highly successful and comprised house inspections, oiling or destruction of mosquito-producing containers, and isolation of yellow fever patients in screened quarters with fumigation of their premises with sulphur or pyrethrum. Havana residents not covering their cisterns were made to pay a US$10 fine. After introduction of the mosquito brigades, yellow fever deaths dropped from an average of 467 per year between 1890 and 1900 to 18 in 1901 [29]. Later the *Aedes* programme was expanded to include *Anopheles* control by drainage, filling or oiling of standing water, ditching in a herringbone pattern, cutting vegetation at edges of ponds or streams, the addition of larvivorous fish or oil to wells, restricting animal grazing during the wet season to avoid creating habitats in flooded footprints, and intermittent short-duration flooding of watercress beds in Havana (versus constant flooding). This resulted in a fall in malaria deaths from 5,643 between 1890 and 1900 to 444 deaths between 1900 and 1910, despite a greatly increased human population [29].

In 1904, Gorgas became Chief Sanitary Officer during the building of the Panama Canal and with the aid of Le Prince eliminated yellow fever and kept malaria at low levels [29]. Control was achieved by screening living quarters, draining or filling standing water, installing drains, and larviciding using oil or Paris Green [30]. Paris Green is not used currently due to high human toxicity and ecological concerns, and we now have much less toxic chemicals that can be applied in a more targeted manner. After these successes, similar campaigns were launched by Joseph White in Havana, Oswaldo Cruz in Rio de Janeiro, and Emilio Ribas in Santos [79]. Successful control was followed by a period of apathy. Complacency was brought to a halt by an epidemic of yellow fever in Rio de Janeiro in 1928 in which population densities of *Ae*. *aegypti* were again at high levels. The Cooperative Yellow Fever Service, a collaboration between the Brazilian Government and the Rockefeller Foundation, was established under the direction of Fred Soper with the aim of eradicating *Ae*. *aegypti* from Brazil. From 1930 to 1934, Soper led a well-organised campaign, with control measures including oiling of water containers and house searches for larvae and adults. Campaigns initiated by the Cooperative Yellow Fever Service also succeeded in eliminating *An*. *gambiae* from the northeast of Brazil in 1942 largely using larviciding with Paris Green, complemented in some areas by house spraying with short-acting pyrethroids [36].

In the late 1800s, colonial expansion in SSA and massive outbreaks of HAT led to a number of scientific missions to study the disease. In 1903, David Bruce and colleagues found that the trypanosome that causes HAT was transmitted by *Glossina palpalis* (now *G*. *fuscipes*) [69]. In colonial times, game destruction and bush clearing were widely practised for tsetse control in many countries. The impetus for game destruction was a natural experiment—a rinderpest epizootic in the 1890s eliminated wild hosts and domestic stock across large parts of east and southern Africa [80]. This led to the disappearance of tsetse from large areas, but as the game populations recovered, so did the tsetse. Game destruction was first introduced in Southern Rhodesia (now Zimbabwe) in 1919, and by 1921, cases of trypanosomiasis were greatly reduced [81]. From 1922 until the early 1980s, game destruction was generally adopted as a tsetse control method [26]. In the 1950s, game destruction in Shinyanga, Tanzania, succeeded in eradicating an isolated tsetse population [25]. Full or partial habitat destruction was also practiced. For example, riverine tsetse species were eradicated from rivers and streams in Ghana and Nigeria by removing short bush and trees with low branches [24]. Despite these successes, the huge environmental impact of bush clearing and game destruction would be unacceptable today.

Traps and targets for tsetse control also started to be developed around this time, as scientists gained an understanding of tsetse behaviour in response to visual (e.g., colour, movement, size/shape) and olfactory cues [27, 82]. For example, in 1909 on the island of Príncipe, estate staff were made to wear squares of black cloth coated with bird lime on their backs, which succeeded in reducing local densities of *G*. *palpalis*. This approach was later incorporated into an island-wide programme (including, also, habitat and host destruction), which eliminated tsetse and lasted until at least 1932 [83, 84]. Early traps developed included the Harris Trap used in Zululand from 1931 onwards against *G*. *pallipides* [83] and the ‘animal’ trap used in West Africa to control *G*. *fuscipes* and *G*. *tachnoides* [85].

From 1942 to 1945, *An*. *gambiae* was eradicated from Upper Egypt (Aswan, Qena, Girga, and Asyût Provinces) through a meticulous campaign of weekly larviciding using Paris Green and, to a lesser extent, house spraying using pyrethrum [43]. Residual treatment of trains, automobiles, and planes was also performed to prevent spread of *An*. *gambiae* to uninfested areas to the north. The number of malaria cases in the region fell from 10,193 cases in 1942 to 59 in 1946.

Prior to the use of residual insecticides, environmental management was also the standard control method against phlebotomine sand fly transmission of *Leishmania* species causing human cutaneous leishmaniasis (CL) and visceral leishmaniasis (VL). Early attempts towards the end of World War I to control sand flies was in response to epidemics of sand fly fever (rather than to leishmaniasis) that disrupted British troops in the Middle East and Mediterranean regions. Based on investigations of local sand fly ecologies and larval development locations, environmental management included the following: pitching camps away from refuse, debris, and loose friable rocks; pitching camps on levelled drained ground; sealing soil cracks with cresol, sand, or sawdust; and treatment with lime—all to render the ground impermeable to ovipositing and emerging sand flies. Additional chemical control included spraying cresol, crude oil, or paraffin or treating with tar around tents and the lower 3 feet (0.9 m) of buildings [86]. Similarly, in India, naphthalene in kerosene with petrol and carbon disulphide was applied 120 yards (110 m) around army barracks, resulting in a 50% reduction in sand flies [86]. Few chemical advances were made against adult sand flies during this period. Bed nets needed to be fine mesh sizes due to the small size of sand flies and were considered too uncomfortable in hot climates. Thus, sand fly control was in existence prior to its definitive incrimination as a vector of *Leishmania*, the result of cumulative experiments between 1904 and 1942 [86–88]. Plastering walls of houses, cattle sheds, and latrines with lime and mud to eliminate cracks used as diurnal resting sites by *Ph*. *argentipes*, a vector in the Indian subcontinent, continues to be part of an integrated approach to sand fly control [89].

### Post–World War II era and the advent of DDT

The first residual insecticide DDT was added to the vector control toolbox in the early 1940s, consequently increasing the popularity of insecticide-based control. World War II had stemmed the supply of pyrethrum (derived from chrysanthemum flowers) from Japan, and alternatives were urgently needed [90]. The first major demonstration of DDT use was at the end of World War II when the allies used the insecticide to control an epidemic of typhus, transmitted by body lice, amongst the populations of war-torn Europe [91].

With the advent of DDT, malaria eradication became a more realistic proposition. In 1947, spurred on by malaria control successes—including the TVA and Malaria Control in War Areas programme to control malaria around military training bases in the southern US [92]—the US National Malaria Eradication Programme was established [44]. This was a joint undertaking by state and local health agencies in 13 southeastern states and the Communicable Disease Center of the US Public Health Service. Indoor DDT spraying, drainage, removal of mosquito larval development sites, and insecticide spraying eliminated malaria transmission, and in 1949 the US was declared free of malaria as a significant public health problem [44]. Support for an eradication approach and IRS using DDT was bolstered by insights from the Ross-Macdonald model, which illustrated that malaria transmission was highly sensitive to reductions in mosquito longevity [93, 94]. In 1955, WHO launched the Global Malaria Eradication Programme (GMEP)—excluding SSA, which they deemed too problematic—with the goal of interrupting transmission through IRS with DDT and other residual insecticides [45, 46]. Environmental management—such as drainage of marshes and housing improvements to prevent mosquito bites—was abandoned, and only later were antimalaria drugs included in the strategy. The GMEP succeeded in eliminating malaria from Europe, North America, the Caribbean, and parts of Asia and South-Central America, primarily where there were more temperate climates, seasonal transmission, and well-functioning control programmes. The northern part of Venezuela was the first to be WHO certified as malaria free in June 1961 through the use of IRS with DDT, sanitary engineering (water management and house improvement), and larviciding [95]. Parasite resistance to drugs, mosquito resistance to insecticides, lack of community participation, and reduced funding led to abandoning the GMEP in 1969 after it was realised that elimination was not possible everywhere with the approach adopted [45].

Also around this time, the Pare-Taveta Malaria Scheme, a pilot project to test the feasibility of malaria eradication in Africa, was initiated [96]. Between 1954 and 1966, the project evaluated the effect of IRS with dieldrin on malaria and mortality in the Taveta subdistrict of Kenya and the Pare district of Tanganyika (now Tanzania). The project succeeded in reducing malaria to low levels, but intensive IRS could not be sustained, and malaria bounced back after the intervention was stopped.

In 1947, Brazil called for elimination of *Ae*. *aegypti* across the whole South American continent, a task that was coordinated by the Pan-American Sanitary Bureau with the continued involvement of Fred Soper [47]. Brazil was encouraged by the success of the Cooperative Yellow Fever Service in northeast Brazil but also understood that *Ae*. *aegypti* could not be eradicated from Brazil unless frontiers and ports were protected from invasion from other countries in the region. Container inspections, oiling of larval development sites, and later perifocal spraying of DDT in water containers and nearby walls succeeded in eradicating *Ae*. *aegypti* from large parts of South America during the 1950s and 1960s. By October 1961, 16 countries in the Western Hemisphere were free of *Ae*. *aegypti* (Bolivia, Chile, Ecuador, Guatemala, Nicaragua, Paraguay, Uruguay, French Guiana, Brazil, Costa Rica, El Salvador, Honduras, Panama, Peru, Canal Zone, and British Honduras) [48]. During the 1940s and 1950s, yellow fever was virtually eliminated in the Americas, but unfortunately, this success became the downfall of the programme. As yellow fever subsided, political support for the eradication campaigns waned despite warnings from Soper about the risk that could result from pulling back the hemisphere-wide *Ae*. *aegypti* control programme [97]. Weak vector control and surveillance meant, as predicted, that *Ae*. *aegypti* gradually returned to many countries, most likely due to re-infestation from countries that had not achieved eradication, including Argentina, French Guyana, the US, Venezuela, and several Caribbean countries.

The advent of DDT and dieldrin also saw the use of residual insecticides for tsetse control. Ground spraying of insecticides onto tsetse resting sites was carried out from the 1950s until the mid-1970s by tsetse control programmes in large parts of East Africa, combined with screening, treatment, and follow-up of patients [98–100]. Aerial spraying was first used in Zululand, South Africa, in the 1950s but was expanded greatly in the 1970s and 1980s. The most successful example of aerial spraying was in the Okavango Delta of Botswana, where it was used from 1973 to 1991 and again in the early 2000s against *G*. *m*. *centralis* succeeding in eliminating tsetse and HAT [25, 51]. Reduced infrastructures for spraying, high costs, and concerns about the environmental impact of DDT and dieldrin, however, resulted in ground and aerial spraying being largely discontinued [69]. The idea of insecticide-treated cattle for tsetse fly control was also developed in the 1940s [101, 102]. Experiments in Tanganyika (now Tanzania) showed that DDT-treated cattle reduced the abundance of *G*. *morsitans* by 99% and *G*. *swynnerloni* by 93% [101]. Insecticides also started to be used in traps and targets. For example, reinvasion of Príncipe by tsetse in the 1950s led to the use of insecticidal ‘animal’ traps between 1956 and 1958, which were successful in eradicating tsetse in Príncipe [103].

Much of the historical control of leishmaniasis has been attributed to IRS using DDT, both targeted specifically against *Leishmania* vectors, or as a beneficial consequence of the GMEP in the 1950s. In China, approximately 530,000 cases of co-circulating zoonotic and anthroponotic *Leishmania* species were reported north of the Yangtze River prior to the creation of the People’s Republic in 1949. The national VL control programme, which started in 1951, included human VL case detection and treatment, IRS of houses and animal shelters using DDT, and elimination or topical deltamethrin treatment of dogs. This campaign reduced the case incidence from 94 per 100,000 population in 1950 to approximately 0.03 per 100,000 population by 1980, with clear effects associated with IRS interventions alone in some regions [49]. In Brazil, where zoonotic VL occurs, the government commission to investigate leishmaniasis and other diseases—created in 1936 and headed by Evandro Chagas—led to the first use of DDT in the 1950s in northeast Brazil. VL case incidence was reduced by 58% in 14 sprayed areas compared to a rise of 12% in 14 untreated areas [104]. Implementation of an integrated approach similar to that adopted in China (without the option of topical insecticide for dogs) led to considerable reductions in the number of cases, though with some reservations about the residuality of DDT on adobe houses [105, 106].

Reductions in *Leishmania* vectors—as well as human incidence, though this is less well reported—have been attributed to the GMEP operating in various countries [107]. In Rio de Janeiro, Brazil, DDT IRS circumstantially led to a reduction from 12.7% prevalence in 1947 to 0.3% in 1953 [108]. IRS spraying during the Indian National Malaria Eradication Programme of 1958 to 1970 apparently resulted in zero cases of VL being reported from the State of Bihar during that time period [109]. Perhaps the more compelling evidence is the rise in VL cases once the GMEP largely ceased in the late 1960s [107, 110]. For example, in the subsequent VL epidemic in Bihar, India, case numbers rose from a few to 40,000 cases by 1978 [109]. Similar rebounds were reported in several countries [107, 110, 111]. Retrospective analysis of household survey data from the Peruvian Andes showed that IRS with DDT from the 1950s until the 1970s resulted in a decrease in CL cases. Cases increased after IRS was discontinued [50].

### GMEP abandoned—What next for malaria?

After the GMEP was abandoned, many countries switched from eradication to malaria control, even though WHO reaffirmed that eradication was still the ultimate goal [112]. During the 1970s and 1980s, malaria was poorly controlled. There were epidemics in the Indian subcontinent (1973–1976) and Turkey (1977) and focalisation of the malaria problem where there was sociopolitical instability or limited socioeconomic development, including countries in SSA, Brazil, and Sri Lanka [46, 113]. The economic crisis in the early 1970s and reorientation of collaborating agencies like the United Nations Children’s Fund (UNICEF) towards general health (rather than malaria) meant less funding for malaria control, oil shortages led to increases in insecticide prices, drug and insecticide resistance were increasing, and operational capacity in programmes was reduced [45]. Also of note was a large WHO-funded research study conducted from 1969 to 1976 in Garki, northern Nigeria, known as the Garki Project [114]. Despite the GMEP not being implemented in Africa, WHO wanted to test whether IRS with propoxur and mass drug administration (MDA) with chloroquine and sulfadoxine-pyrimethamine would interrupt malaria transmission. The measures were successful in reducing parasite prevalence from 80% to 30%, but 1 year after stopping control, malaria rebounded back to pre-intervention levels.

After these setbacks, WHO called for a more tactical approach to malaria control based on the biological, social, ecological, and economic determinants of malaria rather than blanket distribution of insecticide-based vector control and MDA. This approach was endorsed by the World Health Assembly (WHA) in 1978 [115] and further developed at the Seventeenth WHO Expert Committee on Malaria in 1979 [116]. In the early 1990s, WHO devised with member states a new global malaria control strategy, which was endorsed by the WHA in 1993 [117]. The new strategy called for a selective approach in determining whether and where to attempt vector control (and if so, what method to use) and deemphasised IRS, stating that ‘the proper use of insecticides is a complex matter, involving considerable expense and trained personnel and demanding sustained application, usually for many years.’ A renewed focus on research led to new vector control tools becoming available.

In the early 1970s, ITNs emerged as a vector control idea because many communities were already sleeping under untreated bed nets. At this time, synthetic pyrethroids were developed (permethrin, cypermethrin, and deltamethrin), which were safe to use for impregnation of ITNs [118]. Following pioneering trials on ITNs, including those in The Gambia in the 1980s that showed huge impacts on malaria infection and mortality [119–121], WHO recommended the use of ITNs for children and pregnant women [122]. In the early 2000s, the late Chris Curtis and others were strong advocates for ITNs as a public good and for their provision via the public sector with financial assistance from donors [123]. Following several studies that showed a community-level effect of ITNs [124–126] through mass killing of mosquitoes, there was an increasing push for ITNs to be provided to entire communities, rather than just high-risk groups [127]. As a result, the WHO position statement was strengthened in 2007 to recommend the use of long-lasting ITNs (with long-lasting pyrethroid formulations that last for 3 years) distributed either free or highly subsidised and used by all community members [128]. A major stimulus for malaria control at this time was the recognition that malaria restrained economic development and that eliminating malaria in SSA would lead to rapid development [129]. Political will and resources for malaria increased, and in 2007, malaria eradication hit the agenda again following calls by the Bill and Melinda Gates Foundation to eradicate malaria with massive scale-up of existing and experimental interventions [130]. ITN coverage increased rapidly from 2000, predominantly financed through the Global Fund to fight AIDS, Tuberculosis and Malaria [131, 132], and an estimated 50% of the population at risk of malaria of SSA were sleeping under an ITN in 2017 [8]. IRS was used only in specific areas, and therefore only 7% of the population at risk in SSA was covered by IRS in 2017 [8]. Much of the IRS conducted in SSA was funded by the US Presidents Malaria Initiative [133].

Both ITNs and IRS have had considerable public health impact. Between 2000 and 2015, there was a decline in malaria infection by over 50% and an estimated 663 million clinical cases averted in SSA. ITNs were estimated to have been responsible for an estimated 68% of averted cases and IRS for 13% of cases averted [63]. This reliance on insecticide-based malaria vector control has, however, fuelled the development of insecticide resistance [134]. WHO noted that 68 of the 80 malaria-endemic countries that provided data for 2010–2017 reported resistance to at least 1 of the 4 insecticide classes in one malaria vector species, while 57 countries reported resistance to 2 or more insecticide classes [8]. Existing evidence is equivocal as to whether insecticide resistance is adversely impacting malaria control, but it remains a major concern [135–137]. In response to the growing threat of insecticide resistance, a public–private partnership—the Innovative Vector Control Consortium—was established in 2005 to bring new insecticide chemistries to the market, which led to several ongoing market-shaping initiatives on next-generation ITNs and IRS (www.ivcc.com). Despite these efforts, since 2017 WHO World Malaria Reports have highlighted stalling progress against malaria [8, 138, 139]. The reasons for this stagnation include inadequate coverage of ITNs, the need to replace old nets, high rainfall in some parts of SSA, and insecticide resistance. WHO called for increased investment, including filling gaps in coverage of core malaria tools like ITNs [8] and the need to develop additional tools for vector control.

### NTDs—Lagging behind in vector control

Vector control for vector-borne NTDs, including Chagas disease, Gambian HAT, leishmaniasis, LF, and onchocerciasis, took longer to gain traction compared to efforts against anopheline and *Aedes* vectors.

Despite the early discovery by Sir Patrick Manson of transmission of the main LF parasite—*Wuchereria bancrofti*—by mosquitoes in 1878 [67, 140], vector control against LF played a lesser role than for other VBDs. Uniquely among VBDs, LF can be transmitted by 5 genera of mosquito (*Anopheles*, *Aedes*, *Culex*, *Mansonia*, and *Ochlerotatus*), although it typically requires a large number of infective mosquito bites over many years to result in an infectious human host [141]. Although vector control for LF is advocated by WHO [142], much of the focus today is on use of MDA to eliminate microfilariae from the blood of infected humans in order to interrupt mosquito transmission. Despite this, there are several examples from the Pacific region of elimination of LF using vector control alone—including IRS using DDT against *Anopheles* vectors in the Solomon Islands and Papua New Guinea [52–54], as well as sanitation campaigns against culicine vectors in Australia [143]. It is likely that the recent massive rollout of ITNs in SSA has contributed to a decline in LF, although the evidence is equivocal [144–147]. In Papua New Guinea, however, an MDA campaign from 1994 to 1998 reduced transmission, but large declines in mosquito and human infection were not seen until ITNs were introduced in 2009 [148], which was consistent with the notion that combining drugs with vector control would be more effective than either approach alone. Polystyrene beads used in pit latrines against culicine vectors were also shown to augment MDA in India and Zanzibar in the 1980s and 1990s [149–151]. In 1997, WHO called for eradication of LF and 5 other infectious diseases. In 2000 the Global Programme to Eliminate Lymphatic Filariasis (GPELF) was launched, with vector control currently playing a minor role to MDA as a supplementary intervention [152, 153].

Vector control has been integral to the control of onchocerciasis [154], particularly in the Onchocerciasis Control Programme (OCP) in Africa from 1974 to 2002 [55]. Weekly aerial larviciding was conducted from 1976 to 1989 along the rivers of a wide area of west African savanna (700,000 km^2^) where onchocerciasis was endemic [155]. The programme initially covered Benin, Burkina Faso, the northern parts of Côte d'Ivoire, Ghana, southeastern Mali, southwestern Niger, and Togo but was expanded to include additional river basins in Benin, Côte d’Ivoire, Ghana, Guinea, Sierra Leone, and Togo from where *Simulium* were invading [156]. The programme was hugely successful, and the level of onchocerciasis endemicity declined rapidly. Longitudinal entomological surveys at 4 catching points in Burkina Faso showed a decline in annual transmission potential of infective *Onchocerca volvulus* larvae from between 300 and 900 in 1975 to less than 100 by 1982 [155]. The same study reported a reduction in prevalence of human microfilariae infections from around 70% in 1976 to almost negligible levels through to 2000. The absence of—or at least very low levels of—transmission have been maintained since, despite the absence of vector control or ivermectin MDA. Several important lessons were learned from the OCP that can be applied to vector control more widely, including the need to rotate insecticides (implemented following development of resistance to temephos and chlorphoxim) and the value of using a targeted approach to eliminating residual foci based on entomological and epidemiological monitoring [156]. In 1991, the Onchocerciasis Elimination Program of the Americas (OEPA) was launched [157], and in 1995, the African Programme for Onchocerciasis Control (APOC) was established in 19 African countries not included in the OCP [56, 158]. Several of the APOC countries successfully used ground larviciding, although both APOC and the OEPA relied predominantly on community-directed treatment with ivermectin, the use of which was bolstered by the Merck Mectizan donation scheme that began in 1987 [159].

Vector control has also been highly effective against Chagas disease as illustrated by successes of the Southern Cone Initiative (SCI) initiated during 1991 in Argentina, Bolivia, Brazil, Chile, Paraguay, and Uruguay [57–59] and similar initiatives launched in 1997 in Central America and Andean Pact countries. Interventions against the vector *T*. *infestans* focused mainly on IRS, house improvements (replacement of mud walls and floors with cement, and thatched roofs with corrugated metal), and community education. In the 1980s, there were thought to be between 16 and 18 million people infected with *Trypanosoma cruzi*, and the SCI achieved a decline in infestation rate and a sharp decline in the infection rates of children [160]. Uruguay became the first Latin American country to eliminate Chagas disease in 1997 [161], and domestic transmission was effectively eliminated in Chile (1999); Brazil (2006); substantial areas of Argentina, Bolivia, and Paraguay; and parts of Central America [162]. Despite these successes, there are estimated to be still over 6 million people infected with the parasite [6], and continued vigilance is necessary to combat peridomestic and sylvatic vector populations, which can re-invade houses or mediate transmission outside the home [162]. In some regions, successful vector control to maintain low house infestation rates is a prerequisite for human treatment [163].

Vector control continues to play an important role in the fight against leishmaniasis, particularly where sand flies are endophilic [50, 109, 111, 164, 165]. Control is complex, however, due to the numerous aetiologies and transmission cycles, many of which in the Americas are sylvatic, and most *Leishmania* species involve a zoonotic reservoir. To tackle anthroponotic VL, an elimination initiative was launched by India, Bangladesh, and Nepal in 2005 incorporating an IRS component alongside screening and treatment, which was associated with a reduction in VL cases from over 77,000 in 1992 to fewer than 6,000 cases in 2016 [60]. A large cluster randomised trial of ITNs in India and Nepal against anthroponotic VL, however, failed to show effectiveness against infection or disease [166], perhaps due to the vector being more exophagic than expected [167]. In contrast, successful deployment of bed nets reduced anthroponotic CL incidence in a variety of Old World foci [110]. Against zoonotic VL, an alternative insecticide application is deltamethrin-impregnated dog collars, which provide high levels of individual protection against canine infection [168]. Community-level dog collar implementation in Iran reduced human infection incidence by 43% [169] and infantile clinical VL by 50% [168].

Traps and targets for tsetse control also continued to be developed, now incorporating synthetic pyrethroids [82]. In West Africa, the Challier Laveissiére biconical trap was developed and found to catch many more tsetse than the ‘animal’ trap [170]. Deltamethrin-impregnated biconical traps deployed along River Léraba in Burkina Faso in the 1970s rapidly reduced *G*. *palpalis gambiensis* and *G*. *tachinoides* [171]. Different variants of the biconical trap were developed and deployed in different settings, including the pyramidal trap, monoconical and Vavoua trap, bipyramidal trap, and others [27]. Insecticidal targets, panels of insecticide-impregnated cloth, were developed including the R type, which was the first to be widely deployed, and the simplified S type target, which consisted of a rectangle of black cloth flanked by two pieces of black mosquito netting attached to a frame and inserted via a pole into the ground [172]. Pyrethroid treatment of cattle to combat ticks and tsetse was used widely in successful control programmes in Burkina Faso, Tanzania, Zanzibar, and Zimbabwe [173]. The sterile insect technique was used experimentally in Nigeria and Tanzania but only programmatically in Zanzibar, where between 1994 and 1997 tsetse was eliminated [174]. Unfortunately, scaling down of tsetse control campaigns and neglect of surveillance activities led to an increase in HAT cases by the turn of the century. Renewed efforts are underway to eliminate *T*. *b*. *gambiense* and *T*. *b*. *rhodesiense* HAT through reduction of the parasite reservoir using screening and treatment of humans, as well as vector control, including traps and targets for riverine tsetse and insecticide-treated cattle for savannah flies [175, 176]. WHO aims to eliminate HAT as a public health problem by 2020 (with elimination of transmission by 2030) and has developed a roadmap to achieve this goal [177]. Active screening and treatment of human cases provides the mainstay of efforts to achieve the elimination goal. Increased control efforts have coincided with plummeting case numbers from 25,841 cases of *T*. *b*. *gambiense* HAT in 2000 to 2,110 in 2016 and from 709 *T*. *b*. *rhodesiense* HAT cases in 2000 to 54 in 2016 [62]. While active screening and treatment have been responsible for the majority of this decline, in the last decade, new cost-effective methods of controlling riverine species of tsetse—the important vectors of *T*. *b*. *gambiense*—have begun to make an important contribution to the global effort. In particular, ‘Tiny Targets’ are—despite their small size—proving highly effective in reducing the density of riverine species where active screening and treatment alone is not predicted to achieve the elimination goals [178, 179]. Tiny Targets are currently deployed by control programmes in Chad, Côte d’Ivoire, the Democratic Republic of Congo, Guinea, and Uganda.

In Europe, the US, and Australia—where VBDs such as malaria were once endemic—effective disease control programs were replaced by the control of nuisance biting mosquitoes and the intermittent control of epidemics caused by viruses, such as West Nile virus, dengue, and Zika [180]. In Europe, large-scale mosquito control is confined largely to the Rhine Valley, where authorities are conducting larviciding and environmental management [181, 182]. In the US, mosquito abatement districts are present in many states, including large-scale programmes in California and Florida. The abatement district programme has been successful—e.g., in 2018, there were no locally transmitted Zika cases in US states despite importation of cases due to travellers returning from affected areas [61]. The distribution of *Ae*. *aegypti* in Australia is restricted to Queensland, where routine control measures include IRS (targeting the premises of contacts of dengue cases), lethal ovitraps, barrier/harbourage spraying, treatment of containers with residual chemicals, and source reduction [183].

### The growing global threat of *Aedes*-borne diseases

While protozoa and nematodes were the dominant vector-borne pathogens of the 19th to 20th centuries, arboviruses are likely to be most important in the 21st century. *Aedes*-borne viral diseases, including chikungunya, dengue fever, yellow fever, and Zika disease, are a growing threat worldwide [184, 185] due to geographic expansion of vectors and viruses through globalisation and urbanisation [186]. Urbanisation, often unplanned, is typically associated with inadequate housing and lack of basic services, including water and waste management, which creates ideal habitats for expanding *Ae*. *aegypti* populations [187]. *Aedes albopictus*—once confined to tropical forests of Southeast Asia—has increased its geographic range since the mid-1960s and adapted to live in human-made habitats in urban and peri-urban areas [188]. This is exacerbated by a lack of investment in *Aedes* vector control, which in its current form is failing to prevent *Aedes*-borne epidemics. Indeed, as noted by Margaret Chan, Director General of WHO, during her opening address at the 69th WHA in 2016, ‘… Above all, the spread of Zika, the resurgence of dengue, and the emerging threat of Chikungunya are the price being paid for a massive policy failure that dropped the ball on mosquito control in the 1970s.’ Successful *Aedes*-control campaigns of the past were replaced by reactive control during epidemics, which has been largely ineffective. In general, there is a lack of clarity on what vector control methods are effective for *Aedes*-borne diseases because the tools available have not been rigorously assessed against epidemiological outcomes [189]. This is of great concern because in the absence of effective vaccines, programmes that limit contact between humans and vectors and are expedient, comprehensive, and sustained are the most effective method of controlling arboviral diseases [189].

## Discussion

Vector control has been the principal method of preventing VBDs for over 100 years and remains highly effective, when comprehensively applied and sustained. It remains, for several diseases, the only control tool we currently have at our disposal. Key lessons that we can learn from the history of vector control are discussed further as follows and summarised in the ‘Key learning points’.

History shows that complacency and lack of investment in vector control leads to VBD resurgence. A striking example comes from South America, where,—despite achieving eradication of *Ae*. *aegypti* in 16 countries—after control programmes were scaled back the vector reinvaded, flourished, and caused hemisphere-wide epidemics from multiple different viruses [48]. It is estimated that 91% (68/75) of malaria resurgence events from the 1930s to the 2000s were attributed, at least in part, to weakened vector control programmes [190]. Complacency in tsetse control and surveillance led to flareups of HAT in the early 2000s.

Political will and central coordination clearly have important roles to play. For example, the current successes in reducing VL incidence between 2012 and 2017 in the Indian subcontinent [191] are attributed to the advocacy and political support generated with substantial donations from pharmaceutical industry, governments, and nongovernmental agencies [192]. The VL initiative and other programmes—including the Pan American Sanitary Bureau efforts to eliminate *Ae*. *aegypti* from the South American continent, the OCP, and the SCI—all involved strong cross-border collaboration [48, 55, 59]. These large-scale collaborations enabled effective coordination and dialogue, sharing of best practices, and cross-border containment. Effectiveness of this approach is exemplified today by the Elimination8—a collaboration between 8 countries in southern Africa to eliminate malaria (https://malariaelimination8.org/).

It is imperative that political will and investment in vector control is maintained, particularly as we approach elimination targets when the cost per VBD case averted will inevitably increase. Despite this, vector control is chronically underfunded, for research and development (R&D) and at programme level. Investment in vector control R&D is dwarfed by that for drugs, diagnostics, and vaccines [193]. For example, for malaria, only 18 million US$ (3% of total R&D investment) supported vector control in 2014. Although we have seen an increase in investment in dengue vector control R&D from 5 million US$ in 2010 to 21 million US$ in 2014 (an increase from 8% to 25% of total R&D investment), there is negligible or no investment in vector control R&D for other VBDs such as LF. At the programme level, additional funding is needed to increase vector control capacity and build health systems. Programmes lack skilled personnel, in particular public health entomologists and vector control technicians [194]. Surveillance systems and monitoring and evaluation need significant strengthening to allow programmes to target interventions, track progress against programmatic indicators, and make adjustments as needed. Huge funding shortfalls are a roadblock to more effective intervention—even for malaria, which is better funded than other VBDs. Global financing for malaria control and elimination was 2.9 billion US$ in 2015, which represents only 46% of the Global Technical Strategy 2020 annual investment milestone of 6.4 billion US$ [195, 196]. NTDs received only 0.6% of Official Development Assistance in 2012, compared to 6.8% for malaria and 47.2% for HIV/AIDS [197]. At the international level, vector control is losing expertise fast with a massive reduction in the number of entomologists working at WHO’s headquarters in Geneva. To sustain and further the gains already made, political will must be enhanced and investment in vector control dramatically increased.

Throughout the history of vector control, a range of different tools were successfully applied. It is striking, however, that environmental management such as drainage and filling and larviciding come up repeatedly—particularly pre 1940—but were less common thereafter (as noted by Keiser and colleagues for malaria control [22]). Work by Watson and Schwellengrebel, which took an ecological perspective [28, 31], shows that success can be achieved when we have a thorough understanding of the vector and context for transmission as a knowledge base on which to build vector control efforts. Similarly, the development of highly effective baits for tsetse would not have been possible without a rational and detailed understanding of vector behaviour. Unfortunately, the success of this approach is often forgotten in the search for seemingly more easily scalable solutions. Insecticides such as DDT gained favour because they gave a rapid result and were often less labour intensive to implement than environmental management. The availability of residual insecticides initiated a fundamental shift in vector control approaches from locally tailored approaches based on an understanding of the epidemiology and ecological, environmental, economic, and social determinants of VBDs to a focus on deployment of uniform insecticide-based commodities, particularly for malaria. In essence, entomologists stopped being thinkers and became monolithic deployment automatons.

Many of the historical vector control examples involved collaboration with the non-health sector. For example, public–private partnerships such as those in the Zambian Copper Belt and the pioneering work of Celli on improved housing against malaria [37, 38, 74, 77]. This has been largely taken over by health-sector–led vector control. There is huge potential for engagement of the non-health sector for greater resilience against VBDs and increased sustainability of vector control. For instance, farmers can be mandated to dry their fields regularly to reduce *Anopheles* habitats [198], the private sector (e.g., extractive industries) can implement vector control to maintain the health of their workforce and surrounding communities [199], and the housing sector can be engaged to implement housing improvements (e.g., screening) that can dramatically reduce house entry of anopheline and *Aedes* mosquitoes [200].

Development and testing of novel vector control tools is essential. While we are waiting for new tools to come to the market, however, the vector control community should draw upon the full toolbox of interventions that are currently available, including noninsecticidal tools like environmental management. This is particularly important given the changing landscape of VBDs, which mandates a transformation in how we do vector control. For example, insecticide resistance in *Anopheles* and *Aedes* vectors is on the rise and threatens to undermine control of these diseases [134, 201]. Social and environmental change such as urbanisation, climate change, agricultural expansion and intensification, water resource development, deforestation, natural resource exploitation, trade, and population movement are creating enabling conditions for VBD transmission, and current tools do not fully address these VBD determinants [202]. It is increasingly acknowledged that in order to control VBD more effectively and/or drive VBD transmission to zero, multiple interventions need to be applied based on local conditions and needs, and that this may not be achievable with the current tools being used [189, 203–205]. For example, in many settings—even in the presence of universal coverage with core malaria interventions—malaria vectors are able to maintain robust transmission through outdoor biting, feeding on nonhuman animal hosts, and resting outdoors [206]. Environmental management—e.g., house screening against malaria—can also play a role after VBD elimination has been achieved by reducing the risk of reestablishment of transmission.

A criticism of this rationale may be that some of the historic examples, particularly those against yellow fever in South America, involved a ‘military-like’ vector control response in less complex ecosystems that some say would not be feasible today. This is a reasonable point to consider. We argue, however, that a weakness of many of the historic examples was a lack of community engagement in vector control. This could be strengthened, particularly for control of *Aedes* mosquitoes. There are contemporary examples of successful community-based vector control against dengue, HAT, and malaria [207–209].

There is an increasing recognition of the role that vector control can play in disease prevention. For example, the WHO GVCR strategy came about following calls from countries to strengthen vector control in the wake of the Zika epidemic [2]. The GVCR has 4 pillars of action, to (i) strengthen inter- and intra-sectoral action and collaboration; (ii) engage and mobilize communities; (iii) enhance vector surveillance and monitoring and evaluation of interventions; and (iv) scale up and integrate tools and approaches, within the health sector and—where relevant—outside the health sector. Rather than a one-size-fits-all approach, the GVCR advocates for locally appropriate solutions (insecticidal and noninsecticidal) based on solid epidemiological and entomological evidence, which harks back to the approach used by many successful historic vector control programmes. The GVCR will help to galvanise this transformative approach to vector control and should be supported by the entire vector control community.

### Conclusion

Vector control has been shown to be highly effective, historically and presently. Lack of funding and weak programmatic capacity undermine programmes and mean that we are not well equipped to face the pressing new challenges to VBD control, such as environmental change, insecticide resistance, and population growth. There is an urgent need for increased investment in strengthening programmatic capacity for surveillance and control, as well as the development of new vector control tools. We cannot afford to wait until new tools and strategies, such as *Wolbachia* and genetically modified mosquitoes, are available. Instead, we should revisit successful programs from the past and adopt a problem-solving approach that implements tailored vector control solutions drawing upon our entire toolbox of available interventions, including insecticide and non–insecticide-based control methods.

**Key learning points**

- Political will and investment in capacity and capability must be maintained in order to keep VBDs and vector control as public health priorities. If this is not done, disease resurgence is inevitable.
- Train and retain entomologists at all programme levels—a better understanding of the vectors will enable more effective control.
- Encourage cross-border collaboration and regional efforts where possible.
- Expand the toolbox of vector control to include environmental management and larval control and develop the role of sectors outside health to deliver environmental interventions.
- Single vector control tools are unlikely to be sustainable; combined control (including additional vector control tools, vaccines, MDA, and diagnosis and treatment) are more effective and sustainable.

**Top five papers**

1. World Health Organization. Global Vector Control Response 2017–2030. Geneva: WHO; 2017.
2. Watson M. The prevention of malaria in the Federated Malay States. Liverpool: John Murray; 1921.
3. Takken W, Snellen WB, Verhave JP, Knols BGJ, Atmosoedjono S. Environmental measures for malaria control in Indonesia: an historical review on species sanitation. Wageningen: Wageningen Agricultural University Papers; 1990.
4. Soper FL. The elimination of urban yellow fever in the Americas through the eradication of *Ae*. *aegypti*. Am J Public Health. 1963;53:7–16.
5. Hougard JM, Yaméogo L, Sékétéli A, Boatin B, Dadzie KY. Twenty-two years of blackfly control in the onchocerciasis control programme in West Africa. Parasitol Today. 1997;13(11):425–31.
